# MDL: Industrial carbon emission prediction method based on meta-learning and diff long short-term memory networks

**DOI:** 10.1371/journal.pone.0307915

**Published:** 2024-09-06

**Authors:** Feng Li, Meng Sun, Qinglong Xian, Xuefeng Feng

**Affiliations:** 1 Leshan Normal University, Leshan, China; 2 College of Software, Xinjiang University, Urumqi, China; 3 Xinjiang Uygur Autonomous Region Measurement and Testing Research Institute, Urumqi, China; UNITEN: Universiti Tenaga Nasional, MALAYSIA

## Abstract

Greenhouse gas emissions, as one of the primary contributors to global warming, present an urgent environmental challenge that requires attention. Accurate prediction of carbon dioxide (CO2) emissions from the industrial sector is crucial for the development of low-carbon industries. However, existing time series models often suffer from severe overfitting when data volume is insufficient. In this paper, we propose a carbon emission prediction method based on meta-learning and differential long- and short-term memory (MDL) to address this issue. Specifically, MDL leverages Long Short-Term Memory (LSTM) to capture long-term dependencies in time series data and employs a meta-learning framework to transfer knowledge from multiple source task datasets for initializing the carbon emission prediction model for the target task. Additionally, the combination of differential LSTM and the meta-learning framework reduces the dependency of the differential long- and short-term memory network on data volume. The smoothed difference method, included in this approach, mitigates the randomness of carbon emission sequences, consequently benefiting the fit of the LSTM model to the data. To evaluate the effectiveness of our proposed method, we validate it using carbon emission datasets from 30 provinces in China and the industrial sector in Xinjiang. The results show that the average absolute error (MAE), Coefficient of Determination (R^2^) and root mean square error (RMSE) of the method have been reduced by 61.8% and 63.8% on average compared with the current mainstream algorithms. The method provides an efficient and accurate solution to the task of industrial carbon emission prediction, and helps environmental policy makers to formulate environmental policies and energy consumption plans.

## Introduction

Global warming is caused by an increase in the concentration of greenhouse gases in the atmosphere, and this change has serious implications for both human society and economic development. Therefore, the international community has been making efforts to tackle global warming [1]. One of the important measures is to reduce the emission of greenhouse gases such as carbon dioxide [2]. With the rapid development of industrialization and urbanization, China has become the country with the highest CO2 emissions globally. In 2015, China’s industry accounted for about 68% of the country’s energy consumption and 84% of the country’s carbon dioxide emissions [3], so China’s industrial sector has been the focus of the country’s policy to improve carbon and energy efficiency [4]. Xinjiang has 40% of the country’s proven coal reserves, 30% of its oil reserves, and 34% of its natural gas reserves of the same type. At present, Xinjiang’s industrialization development has entered the second half of the initial stage [5], and industry will continue to dominate the national economy for a considerable period in the future. Therefore, accurate prediction of carbon emissions plays an important role in the low-carbon development of industry in Xinjiang’s industrial sector.

For many years, researchers at home and abroad have spared no effort in researching CO2 emission prediction and constantly proposed advanced theoretical methods to improve the prediction accuracy [6]. In the past decades, many classical time series models, such as the autoregressive moving average model (ARMA) [7], the autoregressive integrated moving average model (ARIMA) [8–10], and the generalized autoregressive conditional heteroskedasticity model (GARCH) [11, 12], have been used in the forecasting task of carbon emissions. However, traditional forecasting models are usually constructed based on linear statistical methods, which have advantages for forecasting smooth time series, while carbon emission data are usually non-smooth and non-linear. As a result, linear models perform poorly in real-world carbon emission forecasting. Other researchers have also used the modified gray model (GM) [13–15] to predict carbon emissions, and the results of this method are usually monotonic, less stable, and do not reflect the stochastic variations in CO2 emissions.

With the development of machine learning models [16] and data-driven models [17] in ecosystem conservation, methods such as Support Vector Machines (SVM) [6] and Artificial Neural Networks (ANN) [18–20] have been used for carbon emission prediction. However, machine learning algorithms are difficult to deal with temporal correlation and dependence on time series data, and feature extraction is difficult [21]. In recent years, a large number of deep learning methods have been applied to feature processing of nonlinear carbon emission data. Deep learning methods such as recurrent neural networks (RNN) [22], extreme learning machines (ELM) [23], and deep belief networks (DBN) [24] demonstrated outstanding performance when utilized for the prediction of carbon emissions. LSTM [25] improves the inability of RNN to deal with long-term dependencies by incorporating cellular states and is widely used for carbon emission prediction. Huang et al. [26] used principal component analysis (PCA) and LSTM to predict carbon emissions in China, and experimental results show that the prediction method that combines PCA and LSTM obtains good performance. Zuo et al. [27] proposed an integrated method LSTM-STIRPAT for predicting carbon dioxide emissions in 30 provinces in China. Based on the prediction results, the 30 provinces were categorized into peaked and non-peaked provinces and assessed the driving factors of the different regions. Based on the results of the study, relevant targeted measures to achieve the commitment of China’s peak carbon emissions by 2030 were proposed. Yu et al. [28] validated the STIRPAT model with indicators related to port carbon emissions to estimate the emission trends, and the results showed that the accuracy of the proposed integrated model was improved by 11%. However, deep learning prediction models rely heavily on a sufficient amount of data to estimate the parameters, but the limited data on CO2 emissions poses an obstacle to the practical application of prediction models [29–31]. Comparatively speaking, the use of a small amount of incomplete data to build a precise model for predicting carbon emissions is more relevant for practical application. The purpose of meta-learning is to perform fast and accurate time-series prediction with a small number of samples. A fundamental concept of model-agnostic meta-learning (MAML) [32] is to train the model on multiple tasks with good generalized initial parameters in the hope that many gradient steps can be used to modify the model for the new task. Yao et al. [33] used meta-learning to migrate data from other cities to the target city, and obtained an initialized prediction by learning a model, which might be a suitable fix for the issue of spatio-temporal prediction of urban water quality in the case of insufficient data collection. Shi et al. [34] presented a MAML-based meta-learning method to forecast segmented market demand. Gu et al. [35] extended the MAML algorithm to low-resource neural machine translation (NMT) to learn how to make multilingual high-resource-based tasks solve low-resource linguistic tasks. The model was designed to be a good solution to the problem of spatial and temporal prediction of urban water quality in the case of insufficient data collection. The model is a good solution for the problem of water quality prediction in the case of insufficient data collection.

The primary contribution of this study is the proposal of a meta-learning and differential long- and short-term memory network (MDL) to address the challenge of carbon emission prediction with limited dataset samples. Model agnostic meta-learning (MAML) is also introduced to train a set of initialization parameters that exhibit strong generalization performance for the prediction model, by leveraging prior knowledge while ensuring a certain level of accuracy. Through a series of experiments, we demonstrate that the MAML parameter initialization method outperforms other approaches in carbon emission prediction. In our future work, we plan to incorporate additional feature engineering techniques, such as integrating climate data and economic indicators, to enhance the prediction model’s accuracy.

## Carbon emission prediction method based on meta-learning and diff long short-term memory networks

### Study area and data descriptions

**Fig 1:**
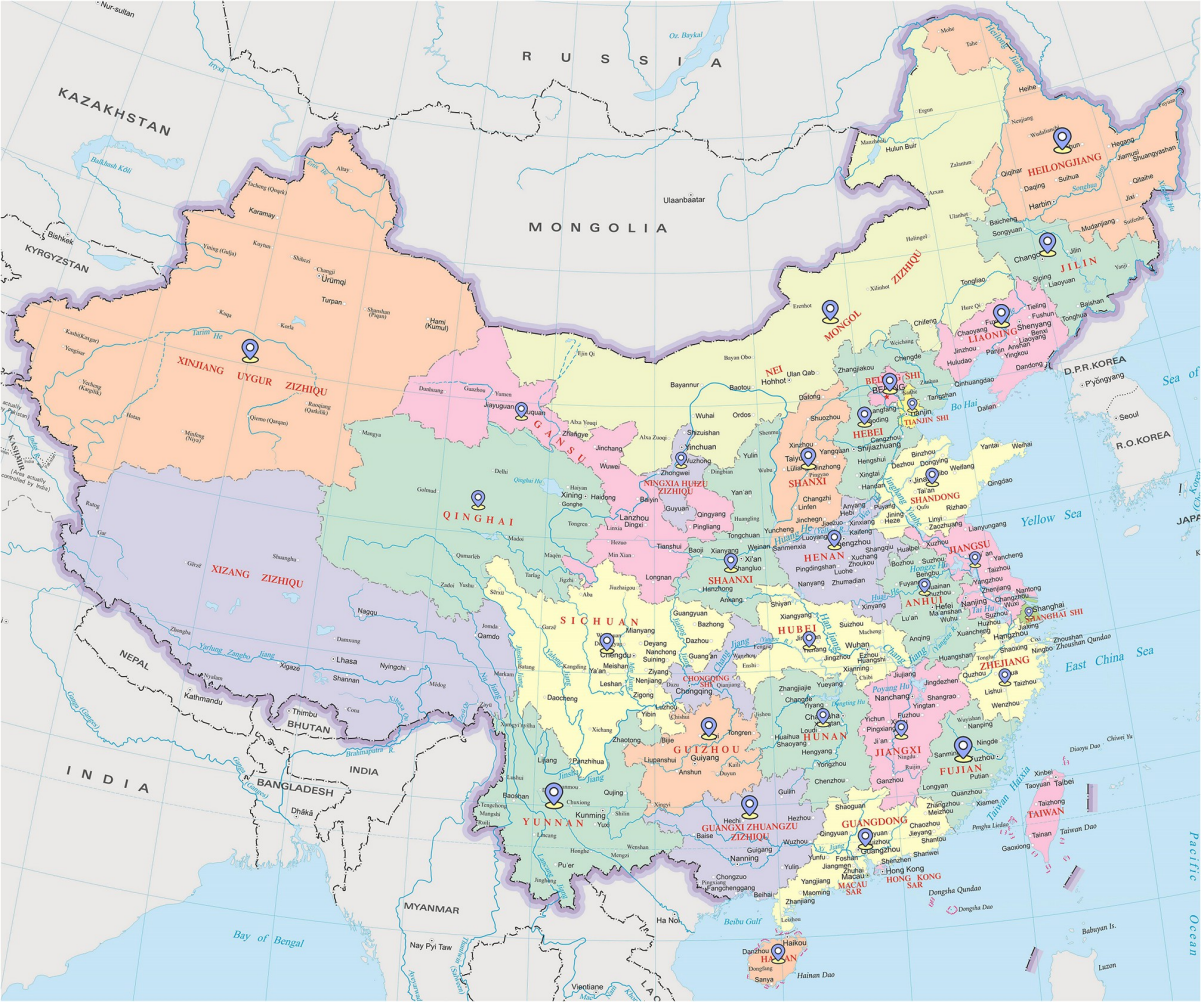
Location of case study.

As shown in Fig 1, we choose China, the country with the highest carbon dioxide emissions, as the main research object, and select the carbon emissions of 30 provinces and cities as well as industries in Xinjiang as the dataset. In order to develop the accuracy of the model, this paper follows the quadratic match-sum method used by Shahbaz et al. [36] to convert annual data from low-frequency data to high-frequency data. Therefore, monthly frequency data from 1995M01–2020M12 were used in this study. Table 1 lists the details of the dataset used, where 312 × 31 indicates that there are 31 tasks with 312 data per task. All data in this paper were obtained from the National Energy Statistical Yearbook (1995–2020) and Xinjiang Statistical Yearbook (1995–2020).

**Table 1:** Basic information on the carbon emissions dataset.

Datasets	Time resolution	Time span	Data volume	Data sources
Industrial Carbon Emissions in 30 Chinese Provinces and Xinjiang	1M	1995–2020	312 × 31	National Energy Statistical Yearbook and Xinjiang Statistical Yearbook

**Fig 2:**
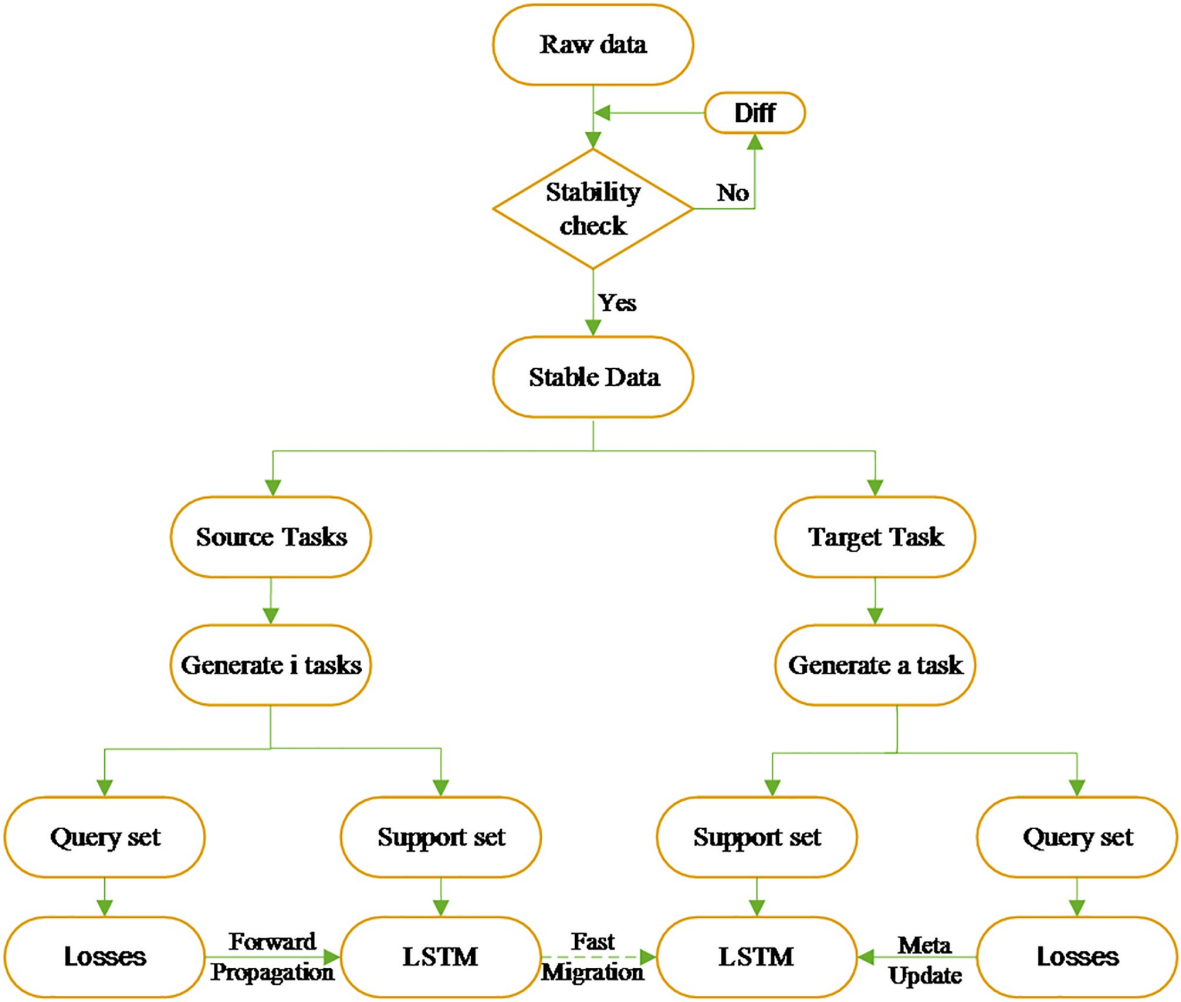
Flowchart of MDL model.

Carbon emission data time series are stochastic time series, which usually exhibit non-stationary characteristics, therefore, the smoothness of the raw carbon emission observation series needs to be assessed [37]. Here, the ADF smoothing test is used to analyze the smoothness of the raw carbon emission data. If it is significant (P<0.05), it means that the original hypothesis is rejected and the series is a smooth time series, and vice versa, it means that the series is an unsteady time series. From Table 2, we can see that the original carbon emission series is a non-stationary time series, at this point, we can calculate its difference based on the original carbon emission series Δ = (1 − *B*)^*d*^ (usually *d* = 1 or 2), so as to obtain the smoothed difference series {Δ*x*_*t*_, *t* = 1, 2, 3, …}. The first-order difference of the original series is After the first-order difference transformation of the original series, the ADF test is performed on the new series to check whether the series is smooth or not [38]. P = 0.007<0.05, which is significant at the level of rejecting the original hypothesis very well, indicating that the series after differencing is a smooth time series. The MDL model structure is shown in Fig 2.

**Table 2:** ADF test results of industrial carbon emission series in Xinjiang.

Variant	Differential order	t-value	P-value	AIC	Threshold		
					1%	5%	10%
Xinjiang industry	0	-0.817	0.814	1974.966	-3.453	-2.871	-2.572
	1	-3.541	0.007***	1968.093	-3.453	-2.871	-2.572
	2	-7.489	0.000***	1972.183	-3.453	-2.871	-2.572

### Long short-term memory network

Long Short-Term Memory, LSTM [25] is an improvement of recurrent neural networks (RNN), which introduces a cell state based on the original model, which adds a long-term memory function.

**Fig 3:**
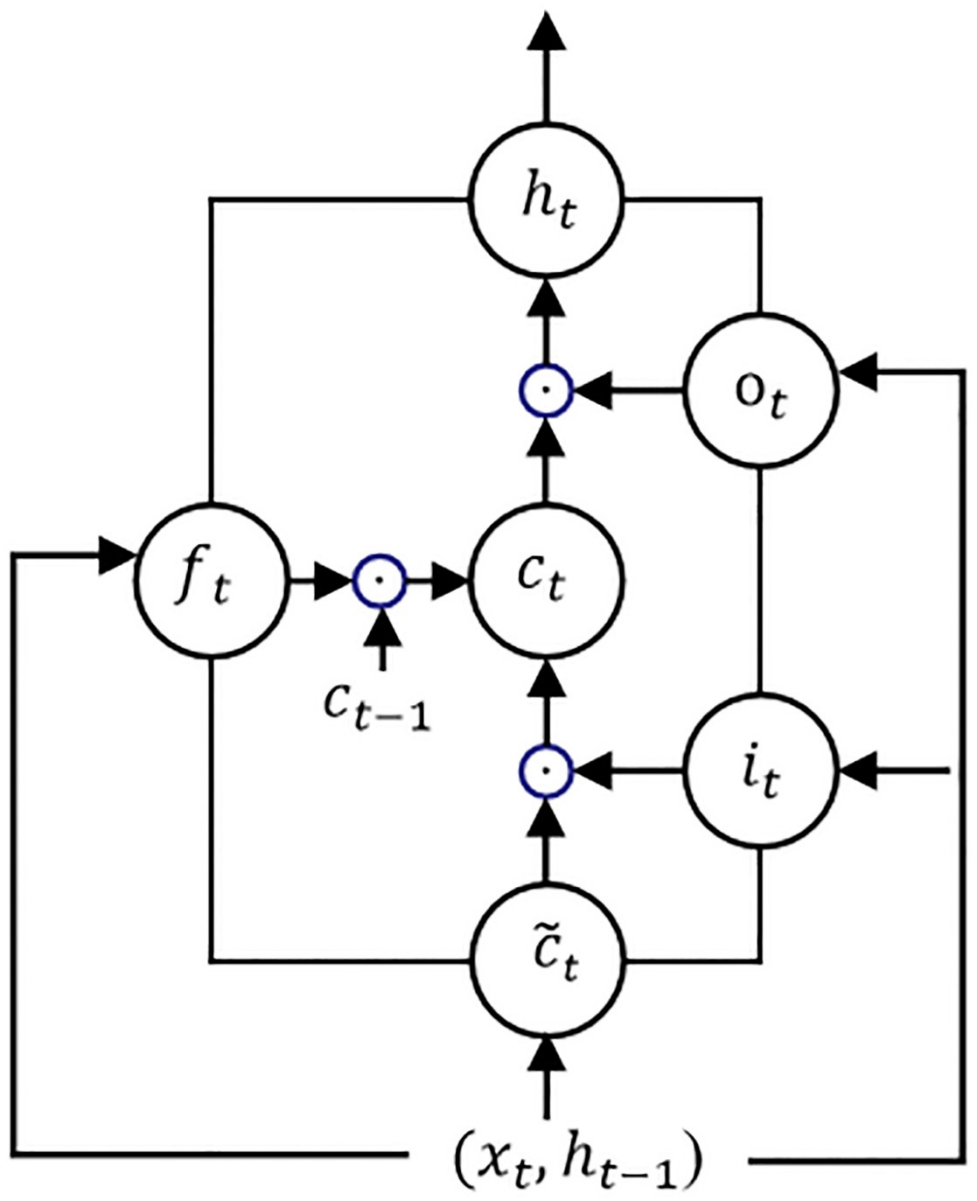
Basic structure of LSTM.

LSTM introduces a gating mechanism, which utilizes “gates” to decide whether information should be passed on or not. Unlike feed-forward neural networks, the input of LSTM consists of the current moment’s data and the previous moment’s LSTM output. In time-dependent problems, the LSTM model shows very good performance, and the basic structure of LSTM is shown in Fig 3.

From the figure, it can be seen that LSTM mainly consists of forget gates, input gates, and output gates.

#### (1) Forget gate

The role of the forget gates is to select invalid information and forget it. It contains two inputs and a neural network layer. The neural network layer processes the input signal of the current moment and the output signal of the previous moment and outputs a result. The calculation is shown in Eq (1):

$$\begin{array}{c} f_{t}=\sigma (W_{f}.[h_{t-1},x_{t}]+b_{f}) \end{array}$$ (1)

where *W*_*f*_ and *b*_*f*_ denote the weight matrix and the bias vector of the forgetting gate, respectively, and *σ*(.) denotes the sigmoid function.

#### (2) Input gate

The role of the input gates is to control the flow of information and to identify the information that needs to be memorized and transmitted or forgotten. It consists of two neural network layers and two input messages. Two of the neural network layers are sigmoid and tanh layers. The input information is the stored information of the previous moment and the input information of the current moment. The specific formula is as follows:

$$\begin{array}{c} i_{t}=\sigma (W_{i}.[h_{t-1},x_{t}]+b_{i}) \end{array}$$ (2)

$$\begin{array}{c} {\tilde{C}}_{t}=\varphi (W_{c}.[h_{t-1},x_{t}]+b_{c}) \end{array}$$ (3)

where *W*_*i*_ and *b*_*i*_ denote the weight matrix and the bias vector of the input gate, respectively. *b*_*c*_ denotes the bias vector of the cell state, and *φ*(.) denotes the tanh function.

#### (3) Update cell state

Updating the cell state means updating the information that needs to be retained and passed on. This information is controlled by forget gates and input gates. Through the forgetting gate and input gate, useless information can be eliminated as well as new information can be introduced. The formula is shown in Eq (4):

$$\begin{array}{c} C_{t}=f_{t}*C_{t-1}+i_{t}*{\tilde{C}}_{t} \end{array}$$ (4)

where *C*_*t*−1_ and *C*_*t*_ denote the cell states at the previous and current moments, respectively, and *f*_*t*_ denotes the output of the forget gate, *i*_*t*_ and ${\tilde{C}}_{t}$ denote the outputs of the two neural network layers in the input gate.

#### (4) Output gate

Output gates determine what information is passed on to the next or output layer. The inputs to the output gate include the output information and cell state from the previous moment and the inputs from the current moment. Based on these inputs, the output gate calculates a value between 0 and 1, which indicates how much information needs to be output at the current moment. This value can be regarded as the “control switch” of the output, if the value of the output gate is closer to 1, it means that the LSTM cell outputs more information. The specific formula is as follows:

$$\begin{array}{c} o_{t}=\sigma (W_{o}.[h_{t-1},x_{t}]+b_{o}) \end{array}$$ (5)

$$\begin{array}{c} h_{t}=o_{t}*\varphi (c_{t}) \end{array}$$ (6)

where *W*_*o*_ and *b*_*o*_ denote the weight matrix and the bias vector of the output gate, respectively, and *c*_*t*_ denotes the cell state at the current moment.

The LSTM network also includes a backpropagation process. In the backpropagation stage, the model backpropagates the error between the output results and the true results along the neural network, updating the weights and biases in the network to improve the accuracy of the model.

### Model-agnostic meta-learning

A well-known algorithm in the field of meta-learning is model-agnostic meta-learning (MAML) [32], which can be combined with any model trained using gradient descent to solve a wide range of machine learning problems such as clustering, dimensionality reduction and regression, etc. Instead of making any a priori assumptions about a specific task, MAML trains a generalized “meta-model” across multiple tasks and adapts it to new tasks. MAML does not require any a priori assumptions about a specific task but rather trains a generic “meta-model” across multiple tasks to adapt to new tasks. Therefore, it can be applied to a wide variety of tasks and can achieve good results with a small amount of data. The main concept of MAML is using meta-learning to train a model, enabling the model to quickly adapt to new tasks on a small number of data samples. The optimization objective of MAML is shown in Eq (7):

$$\begin{array}{c} argmin\sum_{T_{i}\in D_{T}}L_{T_{i}\left(f_{\theta_{i}^{\prime }}\right)=argmin}\sum_{T_{i}\in D_{T}}L_{T_{i}}(f_{\theta -\alpha {▽}_{\theta }L_{T_{i}\left(f_{\theta }\right)}}) \end{array}$$ (7)

where *D*_*T*_ is the set of training tasks, and *T*_*i*_ is the specified training task, and *θ* is the model initialization parameter, and $\theta_{i}^{\prime }$ represents an intermediate result that can be obtained by using the support set of the training set for the first gradient update in the task *T*_*i*_ in which an intermediate result can be obtained by using the support set of the training set for the first gradient update, which is not directly used for the final parameter update, and after obtaining the intermediate results of all the tasks, a second gradient update is performed by using the query set in the training set, and this time the results are directly used for the model parameter update.

The algorithm of MAML is as follows. Overall, the model is mainly divided into two parts: inner and outer learning. Among them, in the process of inner layer learning, the meta-learning algorithm continuously adjusts the parameters of the model to adapt to the learning needs of the new task, and at the same time records the changes of these parameters for use in outer layer learning; in the process of outer layer learning, the meta-learning algorithm utilizes the records of the inner layer learning to direct the learning process of the model, so as to quickly adapt to the learning needs of the new task. First, select *i* tasks *T*_1_, *T*_2_, *T*_3_…,*T*_*i*_ from the source task dataset *S* to form a task set *T*. For each task *T*_*i*_, divide the training set and test set within the task, the training set within the task is called the support set $T_{i}^{S}$, and the test set is called the query set $T_{i}^{Q}$. In the inner loop, the model first randomly initializes the parameter *θ* and then calculates the loss of the support set based on that parameter and the support set of the training set and updates its unique copy of the parameter $\theta_{i}^{\prime }$ The model then saves it for subsequent use while releasing the above loss. In the outer loop, the model loads the parameters $\theta_{i}^{\prime }$, and sums the loss of the query set based on these parameters and the sample query set data from the training set, and uses the result of this sum to update the model’s initialization parameters *θ*.

**Algorithm 1** MAML

**Require:** source task dataset *S* and the target task *T*; *α*, *β*: step size hyperparameters;

 1: Randomly initialize *θ*

 2: **while** not done **do**

 3:   sample batch of tasks *T*_*i*_ ∈ *S*

 4:   **for** all *T*_*i*_ **do**

 5:    Calculate the loss for each task *T*_*i*_: $L_{T_{i}^{s}}(f_{\theta })$

 6:    Gradient descent based on the calculated obtained losses $\theta_{i}^{\prime }=\theta -\alpha {▽}_{\theta }L_{T_{i}^{S}}(f_{\theta })$;

 7:   **end for**

 8:   Update model parameters $\theta \leftarrow \theta -\beta {▽}_{\theta_{i}^{\prime }}\sum_{i=1}^{k}L_{T_{i}^{Q}}(f_{\theta_{i}^{\prime }})$;

 9: **end while**

## Results

### Dataset construction and stationary analysis

Carbon emissions prediction is a simulation methodology that predicts future emissions based on historical data. In order to calculate carbon emissions from industrial sectors in 30 Chinese provinces and Xinjiang, standardized end-use energy consumption is used to calculate final carbon emissions by excluding the amount of energy used for processing and conversion as well as losses according to the energy balance. At present, the main methods for calculating carbon dioxide emissions are energy based carbon source estimation, with the most widely used being the carbon emission coefficient method provided in Chapter 6 of Volume 2 Energy of the IPCC Guidelines for National Greenhouse Gas Emissions Inventory [39]. This paper refers to the IPCC Guidelines and measures carbon dioxide emissions based on primary energy consumption, with the following formulas:

$$\begin{array}{c} I=\sum_{i=1}^{n}\;F_{i}\times E_{i}=\sum_{i=1}^{n}\frac{O_{i}\times VCV_{i}\times CC_{i}}{CF_{i}}\times E_{i}\times \frac{44}{12} \end{array}$$ (8)

where *I* is the amount of CO_2_ emissions, and *i* is the type of primary energy source, the *E*_*i*_ and *F*_*i*_ denote the energy *i* consumption and carbon emission factor, the *CF*_*i*_ and *CC*_*i*_ denote the energy *i* coefficients of standardized coal and carbon content per unit of calorific value of the energy sources, the *NVC*_*i*_ and *O*_*i*_ denote the average low-level heat production and carbon oxidation rate of the energy source *i*, and 44/12 is the mass fraction of elemental carbon in CO_2_. The discounted standard coal factor and average low-level heat generation for the eight categories of primary fossil energy are from the China Energy Statistics Yearbook 2019 [40], and the default carbon oxidation factor and default carbon content are from the IPCC Guidelines for National Greenhouse Gas Emissions 2006. Table 3 provides detail. A small fraction of the carbon in a fuel is not oxidized during combustion. Although this fraction is typically small (99–100% of the carbon is oxidized), the factors in Table 3 are derived from the assumption of 100% oxidation. In other words, the proportion of carbon oxidized is assumed to be 1 when generating the default CO2 emission factor [41].

**Table 3:** Emission factors for different energy varieties.

Type of energy	Average low-level heat generation (kJ/kg)	Default carbon content (kgc/GJ)	Default Carbon Oxide Factor B	Conversion factor of standard coal (kgce/kg)	Carbon emission factor (kgc/kg)
coals	20934	25.8	1	0.7143	0.7559
coke	28470	29.2	1	0.9714	0.8550
crude oil	41868	20	1	1.4286	0.5857
Gasoline	43124	20.2	1	1.4714	0.5920
Kerosene	43124	19.5	1	1.4714	0.5714
diesel fuel	42705	20.2	1	1.4571	0.5921
fuel oil	41868	21.1	1	1.4286	0.6185
petroleum	38979kJ/m3	15.3	1	1.33kgce/m3	0.4483

### Evaluation indicators

To ensure the scientific validity of the experimental results and the diversity of performance indicators, this paper will be more convincing to assess the performance of the proposed model from multiple perspectives. The Symmetric Mean Absolute Percentage Error (SMAPE), RMSE, and MAE are used to evaluate the model’s prediction accuracy [42, 43].

$$\begin{array}{c} \text{RMSE}=\sqrt{\frac{1}{N}{\sum_{i=1}^{N}(Y_{i}-{\hat{Y}}_{i})}^{2}} \end{array}$$ (9)

$$\begin{array}{c} \text{MAE}=\frac{1}{N}\sum_{N}^{i}|Y_{i}-{\hat{Y}}_{i}| \end{array}$$ (10)

$$\begin{array}{c} \text{SMAPE}=\frac{200\%}{N}\sum_{i=1}^{N}\frac{\left|Y_{i}-{\hat{Y}}_{i}\right|}{\left|Y_{i}|+|{\hat{Y}}_{i}\right|} \end{array}$$ (11)

Where *Y*_*i*_ is the actual value and ${\hat{Y}}_{i}$ is the predicted value. *N* is the number of samples. The smaller the value of RMSE, MAE, and SMAPE, the higher the prediction accuracy.

### Experimental settings

In this section, the time series data for carbon emissions over 30 provinces in China from 1995M01 to 2020M12 are regarded as the source task dataset *S*, and the carbon emission time series data of the industrial sector in Xinjiang are regarded as the target task *T*. The training and testing for the machine learning models are implemented in PyTorch version 1.9.0, DGL version 0.7.2 with CUDA version 10.2, scikit-learn version 0.24.1 and Python 3.7. The parameters *θ* are used for prediction of the target task by learning them on the source task dataset. The historical data is divided into a training set of 50% and a test set of 50%. In order to explore the robustness and effectiveness of the MDL model, we set three different prediction ranges *M*= 10, 20, 30. Time-series prediction is usually regarded as a regression task, so when training the model, we usually choose the MSE as the loss function.

$$\begin{array}{c} Loss=\frac{1}{H}\sum_{i=1}^{H}(Y_{T+i}-{\hat{Y}}_{T+i}{)}^{2} \end{array}$$ (12)

To progressively demonstrate the efficacy of the suggested approach, MDL is contrasted with multiple approaches:

LSTM [44]: This network architecture is characterized by its unique gating mechanism that captures the temporal dependencies between timing data very well, with a strong long-term memory capability. The performance of LSTM in the task of timing prediction is well recognized in this literature.

MAML_LSTM: Long short-term memory networks incorporating a meta-learning framework.

D_LSTM: The raw carbon emission sequence is used as an input to the LSTM model after differencing.

For LSTM and D_LSTM, the model performs gradient descent on the training set of the target task to minimize the loss function; however, since the training set of the target task is very small, the model parameters are updated only once after the model calculates the loss for all the data in the training set during the training process. When training the LSTM and D_LSTM, the LSTM hidden units are set to 100 and dropout is used to avoid overfitting; we set the maximum number of iterations to 500 and use the Adam optimizer to train the model, where the learning rate is 0.03.

For MAM_LSTM and MDL, cross-task training on the source task dataset *S* allows the model to learn transferable meta-knowledge, which not only helps it to converge quickly on the target task, but also alleviates the overfitting problem *S*. When training the MAML_LSTM and MDL models, the task network update frequency refers to the number of parameter updates for a single task sample; the Adam optimizer is used, where the learning rates of the outer and inner layers of the loop are set to 0.03 and 0.04, respectively, for better performance. The hyperparameter settings for the training of each model are shown in Table 4.

**Table 4:** Hyperparameter settings during model training.

	LSTM	D_LSTM	MAML_LSTM	MDL
learning rate	0.03	0.03	-	-
Number of iterations	500	500	500	500
Endogenous learning rate	-	-	0.04	0.04
Outer learning rate	-	-	0.03	0.03
Sample size (statistics)	-	-	5	5
Number of mission network updates	-	-	10	10

## Analysis of experimental results

### Analysis of the results of the prediction of industrial carbon emissions in Xinjiang

According to the experimental setup, we trained the models under three different prediction ranges *M*= {10, 20, 30}. Table 5 shows the RMSE, MAE, and SMAPE of the four models on the Xinjiang industrial carbon emission dataset under different prediction ranges. Taking the prediction range M = 10 as an example, it can be seen from the table that the RMSE, MAE and SMAPE of the LSTM model are only 0.683, 0.050, and 0.063, respectively; after incorporating the MAML framework, the RMSE, MAE, and SMAPE were reduced by 35.1%, 27.5% and 26.9%, respectively, indicating that the method of obtaining initialization parameters after MAML model training is better than the prediction effect of LSTM, which well solves the problem of less carbon emission data. The RMSE, MAE, and SMAPE of the original carbon emission series smoothed and then input to the LSTM model are also reduced by 67.2%, 63.5%, and 40.8% respectively compared with LSTM, indicating that the noise can be suppressed after differencing, and the data curve becomes relatively smooth, which is favorable to the fitting of the series by LSTM. The MDL model combines the advantages of MAML_LSTM and D_LSTM, and its RMSE, MAE, and SMAPE, respectively, are reduced by 94.8%, 94.2%, and 91% compared to LSTM, and RMSE, MAE, and SMAPE are both reduced by 92.1% and 87.7% compared to MAML_LSTM, and 84.3%, 84.1%, 84.1%, and 84.8% respectively compared to D_LSTM, 84.1% and 84.8%, respectively. From the results of R^2^, it can be seen that the model of MDL has the best interpretability to the data.

**Table 5:** Prediction results of different methods for industrial carbon emissions in Xinjiang.

Range of projections	Model	R^2^	RMSE	MAE	SMAPE
M = 10	LSTM	0.3724	0.0683	0.0501	0.0632
	MAML_LSTM	0.3625	0.0443	0.0363	0.0462
	D_LSTM	0.4748	0.0224	0.0182	0.0374
	MDL	**0.5635**	**0.0035**	**0.0029**	**0.0057**
M = 20	LSTM	0.3074	0.1121	0.0875	0.1041
	MAML_LSTM	0.4983	0.1148	0.0874	0.1031
	D_LSTM	0.4432	0.1028	0.0452	0.1017
	MDL	**0.5388**	**0.1028**	**0.0382**	**0.0852**
M = 30	LSTM	0.3427	0.1376	0.1135	0.1370
	MAML_LSTM	0.3028	0.1321	0.0981	0.1137
	D_LSTM	0.4148	0.1717	0.0659	0.1490
	MDL	**0.5234**	**0.1044**	**0.0466**	**0.1071**

Table 6 compares the difference between the number of model iteration training for LSTM and MAML_LSTM for three different prediction ranges, where the number of model iteration training refers to the number of iterations of the model on the training set when the model obtains the optimal result on the test set. Here the number of iterative training times for MAML_LSTM refers to the number of iterations on the support set on the target task. From the table, it can be seen that the training number of MAML_LSTM has decreased compared to LSTM, which is because meta-learning can obtain meta-knowledge that can be migrated for use by training on a large number of source task datasets, obtaining more sensitive model parameters, a smaller number of training iterations, and mitigating the overfitting problem of the long short-term memory network-based models for the carbon emission prediction scenarios.

**Table 6:** Number of model training iterations.

Model	M = 10	M = 20	M = 30
LSTM	109	233	429
MAML_LSTM	56	76	84

### Analysis of carbon emission prediction results for 30 provinces in China

To further validate the effectiveness of MDL, we validate it on the carbon emission dataset of 30 Chinese provinces from 1995M01–2020M12. Specifically, we consider the carbon emission data of 30 provinces as 30 tasks, and for each task, set three different prediction ranges, i.e., *M* = {10, 20, 30}, to explore the generalization and robustness of the MDL model. The LSTM and D_LSTM have the same experimental setups as in the previous section, and for the MAML_LSTM and the MDL, each task can be used as the target task *T* and all other tasks belong to the source task dataset *S*, then |*S*| = 29. To ensure the fairness of the experiments, the experimental setup of MAML_LSTM and MDL is used for all of this section.

**Fig 4:**
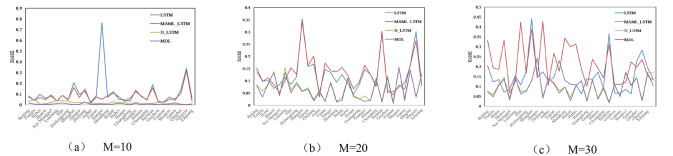
Comparison of performance of different models on RMSE.

**Fig 5:**
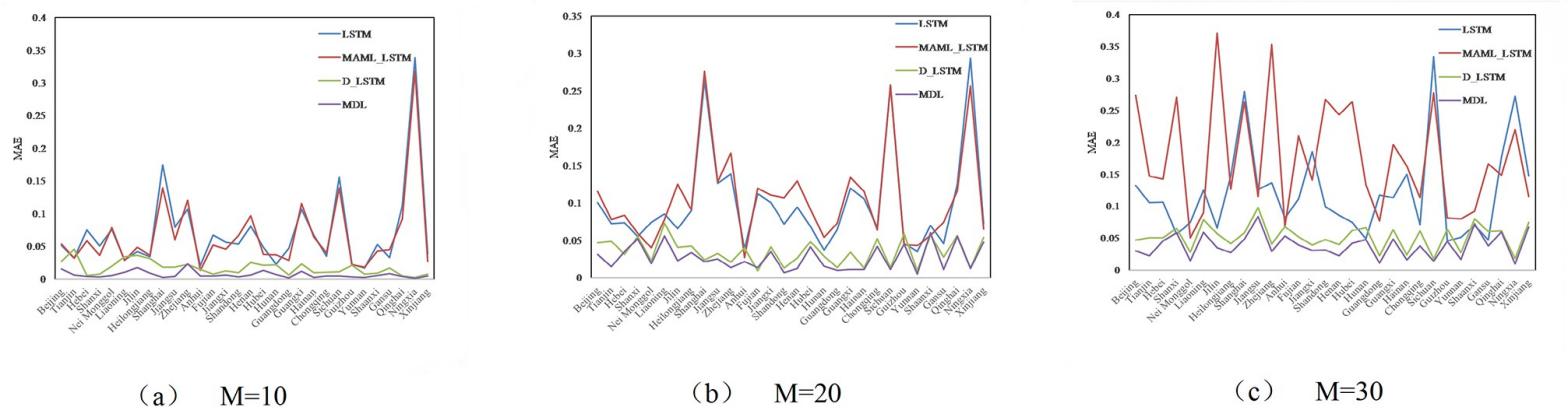
Comparison of performance of different models on MAE.

**Fig 6:**
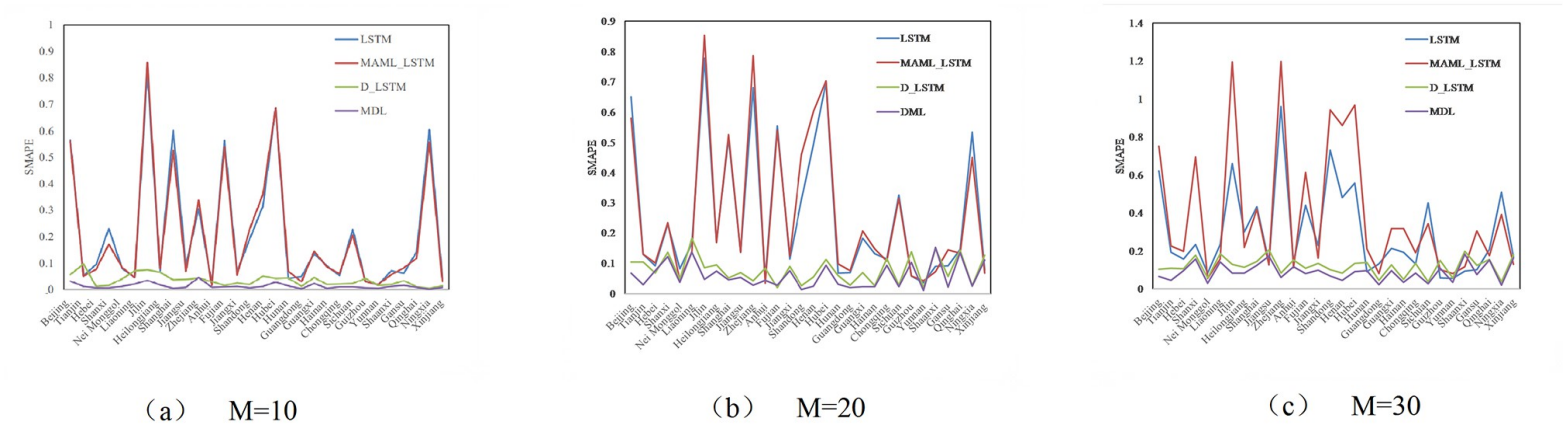
Comparison of performance of different models on SMAPE.

Figs 4–6 compare the RMSE, MAE, and SMAPE of the four models under three different prediction ranges of *M*={10, 20, 30}. Observing Figs 4–6, under three different prediction ranges, the MDL model still outperforms the base model as a whole, although the performance of the MDL model deteriorates in a few tasks. This indicates that the MDL model has stronger generalization ability and robustness, and can be better adapted to complex timing prediction tasks.

Table 7 shows the average values of R^2^, SMAPE, MAE, and RMSE of each model for the 30 tasks under the prediction range of *M*= {10, 20, 30} in order to quantitatively compare their performance performances. Taking the prediction range *M*=10 as an example, Table 7 demonstrates that the average R^2^, the average RMSE, average MAE, and average SMAPE of the MAML_LSTM model are reduced by 27.7%, 6.41% and 2.25%, respectively, compared to the single LSTM, which indicates that the MAML parameter initialization method reduces the prediction error. D_LSTM model’s average RMSE, average MAE and average SMAPE are reduced by 75.9%, 76.3% and 83.8% respectively compared to single LSTM, indicating that the prediction performance can be enhanced by smoothing the sequences. MDL has better performance than LSTM, MAML_LSTM, and D_LSTM, and its average RMSE, average MAE, and average SMAPE are reduced by 93.6%, 91% and 93.8% compared to single LSTM, 91.1%, 90.3% and 93.8% compared to MAML_LSTM, and 63.8%, 61.6% and 61.8% compared to D_LSTM. reduced by 63.8%, 61.8% and 62.5%. It can be seen that the MDL model with the simultaneous introduction of differential and MAML frameworks can further reduce the prediction error and well solve the problem of non-stationary carbon emission data and small data volume.

**Table 7:** Mean values of evaluation indicators of each model under different prediction ranges.

Range of projections	Model	R^2^	RMSE	MAE	SMAPE
M = 10	LSTM	0.3825	0.1105	0.0707	0.2123
	MAML_LSTM	0.3706	0.0799	0.0662	0.2075
	D_LSTM	0.4646	0.0192	0.0167	0.0343
	MDL	**0.5472**	**0.0071**	**0.0064**	**0.0129**
M = 20	LSTM	0.3796	0.1237	0.0989	0.2588
	MAML_LSTM	0.3081	0.1219	0.0959	0.2690
	D_LSTM	0.4433	0.0687	0.0349	0.0780
	MDL	**0.5259**	**0.0637**	**0.0262**	**0.0583**
M = 30	LSTM	0.3428	0.1543	0.1213	0.2995
	MAML_LSTM	0.3025	0.1407	0.1154	0.2880
	D_LSTM	0.4289	0.0987	0.0522	0.1185
	MDL	**0.5108**	**0.094**	**0.0386**	**0.0881**

**Fig 7:**
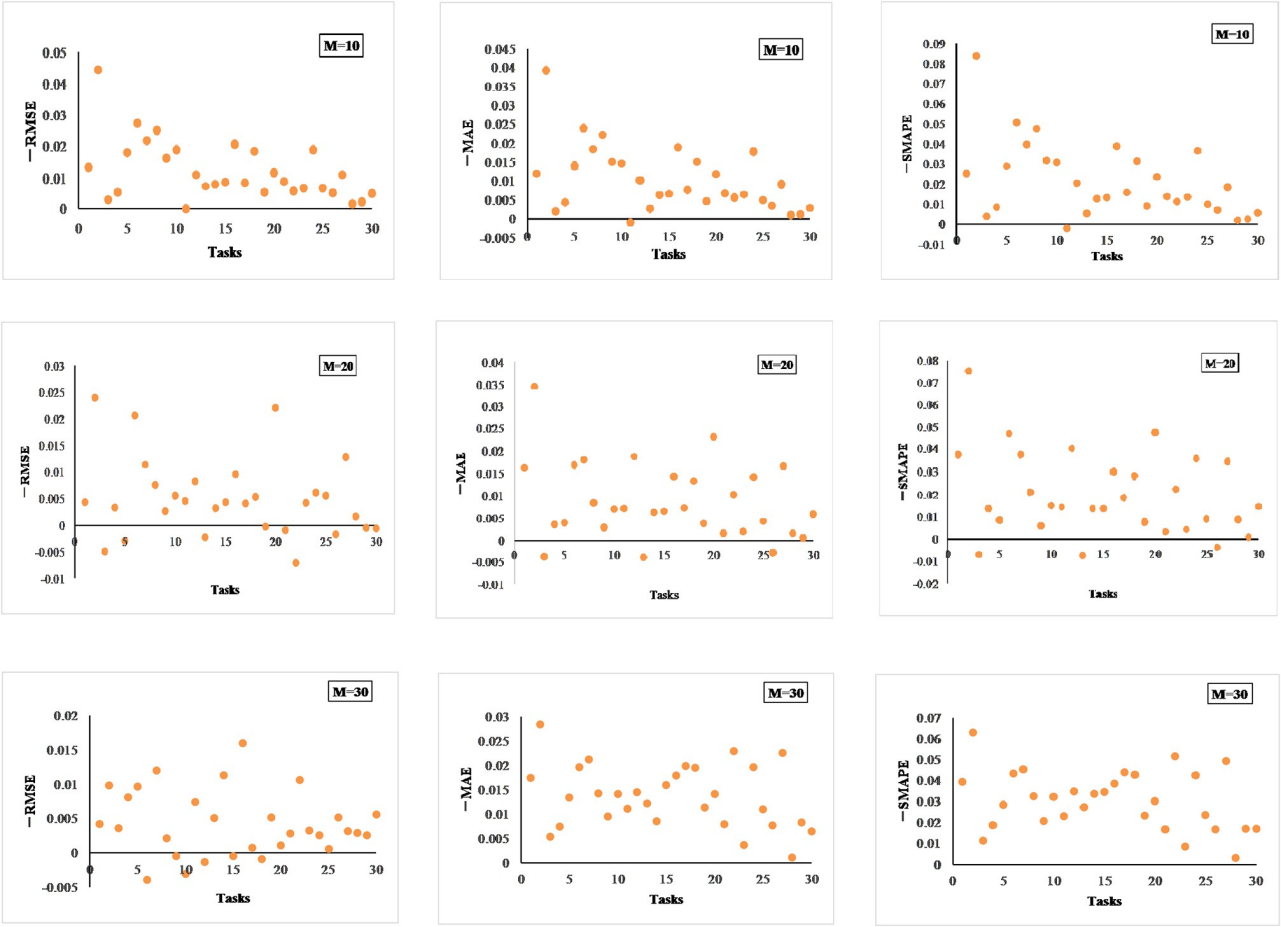
Performance comparison between MAML_LSTM and MDL.

Fig 7 compares the differences between D_LSTM and DML in the evaluation metrics RMSE, MAE, and SMPAE for three different prediction ranges; the horizontal represents the different tasks and the vertical represents the differences in each metric. For example, in Fig 7, -RMSE represents the RMSE of DML subtracted from the RMSE of MAML_LSTM. A value of -RMSE is greater than zero, and the predictive performance of DML is better than that of D_LSTM. Observing Fig 7, it can be found that, under the three different prediction ranges, DML has significant performance improvement, and only on a small number of tasks there is a performance degradation. Table 8 quantitatively reflects the experimental results of Fig 7, from which it can be seen that the tasks with performance degradation in RMSE increase as the prediction range increases, whereas the metrics of MAE and SMAPE do not show performance degradation as the prediction range increases.

**Table 8:** Percentage of performance increase or decrease in each metric for D_LSTM vs. DML.

Range of projections	RMSE	MAE	SMAPE
M = 10	100%	97%	97%
M = 20	76%	90%	90%
M = 30	80%	100%	100%

**Fig 8:**
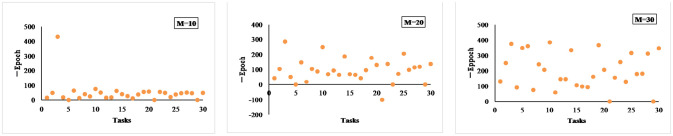
Comparison between LSTM and MAML_LSTM in terms of number of iterations.

Fig 8 compares the difference between the number of model iteration training for LSTM and MAML_LSTM for three different prediction ranges, where the number of model iteration training refers to the number of iterations of the model on the training set when the model goes to the optimal result on the test set. Here the number of iterative training times for MAML_LSTM refers to the number of iterations on the support set on the target task. As can be seen in Fig 8, MAML_LSTM can achieve convergence with fewer training batches. This means that MAML_LSTM can achieve the desired results with smaller batches and datasets. This is because MAML performs outer layer learning by summing the final updated parameters over the test set on each task, summing all the losses before performing stochastic gradient descent optimization, which allows the initialization parameters to adapt to each task as much as possible.

## Conclusion

In this paper, we propose a method MDL to predict carbon emission based on meta-learning and diff long short-term memory networks. The initial time series is transformed by MDL into a comparatively smooth series, which is beneficial for LSTM to fit the data. Considering 30 provinces in China as the source task and the industrial carbon emission data in Xinjiang as the target task, the meta-learning framework MAML is introduced to solve the carbon emission prediction problem of the industrial sector of Xinjiang under the situation of limited data volume. Emission prediction problem in the Xinjiang industrial sector with limited data volume, which lays the foundation for formulating appropriate carbon emission strategies and corresponding policies. We also validate the effectiveness of the proposed method in this paper on carbon emission datasets from 30 provinces in China. The experimental results show that MDL outperforms LSTM, LSTM_MAML, and D_LSTM on most tasks, illustrating the effectiveness of the MAML initialization parameter method and the superior performance of the diff long short-term memory network. In addition, the methods in this paper can help policymakers develop more scientific energy policies to reduce carbon emissions and promote sustainable development.

Future research could consider using other neural network models combined with meta-learning frameworks for performance evaluation of models for predicting carbon emissions. Other countries with high CO2 emissions could also adopt the process of this study’s methodology to develop carbon emission prediction models with better generalization performance to guide decision-making in formulating environmental policies.

## Supporting information

S1 Data There are two files (train_data.csv and test_data.csv) in this zip file, training data and test data respectively. (ZIP)
